# Luminous, relativistic, directional electron bunches from an intense laser driven grating plasma

**DOI:** 10.1038/s41598-022-21210-7

**Published:** 2022-10-07

**Authors:** Amit D. Lad, Y. Mishima, Prashant Kumar Singh, Boyuan Li, Amitava Adak, Gourab Chatterjee, P. Brijesh, Malay Dalui, M. Inoue, J. Jha, Sheroy Tata, M. Trivikram, M. Krishnamurthy, Min Chen, Z. M. Sheng, K. A. Tanaka, G. Ravindra Kumar, H. Habara

**Affiliations:** 1 Tata Institute of Fundamental Research, 1 Homi Bhabha Road, Colaba, Mumbai 400005 India; 2 Graduate School of Engineering, Osaka University, Suita, Osaka 5650871 Japan; 3 Key Laboratory for Laser Plasmas (Ministry of Education), School of Physics and Astronomy, Shanghai Jiao Tong University, Shanghai, 200240 China; 4 Faculty of Science and Engineering, Setsunan University, Neyagawa, Osaka 5728508 Japan; 5 Collaborative Innovation Center of IFSA (CICIFSA), Shanghai Jiao Tong University, Shanghai, 200240 China; 6 Department of Physics, SUPA, University of Strathclyde, Glasgow, G4 0NG UK; 7 Extreme Light Infrastructure: Nuclear Physics, 30 Reatorului, 77125 Magurele, Bucharest, Romania; 8 Department of Physics, School of Basic and Applied Sciences, Central University of Tamil Nadu, Thiruvarur, 610005 India

**Keywords:** Plasma physics, Laser-produced plasmas, Ultrafast lasers, Micro-optics

## Abstract

Bright, energetic, and directional electron bunches are generated through efficient energy transfer of relativistic intense (~ 10^19^ W/cm^2^), 30 femtosecond, 800 nm high contrast laser pulses to grating targets (500 lines/mm and 1000 lines/mm), under surface plasmon resonance (SPR) conditions. Bi-directional relativistic electron bunches (at 40° and 150°) are observed exiting from the 500 lines/mm grating target at the SPR conditions. The surface plasmon excited grating target enhances the electron flux and temperature by factor of 6.0 and 3.6, respectively, compared to that of the plane substrate. Particle-in-Cell simulations indicate that fast electrons are emitted in different directions at different stages of the laser interaction, which are related to the resultant surface magnetic field evolution. This study suggests that the SPR mechanism can be used to generate multiple, bright, ultrafast relativistic electron bunches for a variety of applications.

## Introduction

High energy particle bunches are a major tool in modern science and technologies^1–4^. In recent years ultra-short, high intensity laser pulses have emerged as compact drivers for high energy particle beams from various targets in different phases of matter^1–4^. It has however become obvious that further progress in laser driven particle beams requires smart manipulations of laser pulses and/or target properties. One nice example, is the invocation of the enhanced surface electric field created when such lasers impinge on a solid target that has modulated surface structures^5–24^. The structures can either be randomly oriented as in nanoparticle^5^ or nanotube^6,7^ coatings, or somewhat defined like aligned structures like nanorods^8^, nanotubes^9^, microspheres^10,11^ or well-defined modulations as in grating structures^11–26^. Although all these structures^5–30^ contribute to the enhancement of the laser absorption because of increase of interaction area and effects like the lightning rod^31^, grating structures^11–30^ were found to generate hotter and more copious flux of electrons via resonant excitation of surface plasmon, the so-called surface plasmon resonance (SPR).

The extrapolation of such behaviour to higher intensities is however still to be established because of potential damage to the grating structures by the rising edges and precursors of the ultra-intense laser pulses, which has so far been limited to moderate intensities^14–16^. Even after observation of higher energy of accelerated protons^17^ and efficient conversion of laser energy to electrons^32–34^ (using two-dimensional particle-in-cell simulations) on a grating target at relativistic intensity, it is still unclear whether SPR or local field concentration (similar to nano-size structure) governs the absorption mechanism. The Brunel absorption (vacuum heating)^35^, a well-known mechanism for absorption at high intensity laser pulses, has an optimum incident angle, and is governed by the plasma scale length at target front^36^. Such angular dependence also complicates the observation of SPR. Interestingly, periodically modulated/grating targets are found to be efficient for X-rays^12^, electron emission from target surface and front side^15,16,27^, and proton acceleration^17^. Also, these targets are explored intensively to generate higher harmonic^18–21,28^, and steady magnetic fields^26^. A recent summary of the subject can be found in Ref.^22^.

Here we demonstrate efficient coupling of relativistic intensity (~ 10^19^ W/cm^2^) femtosecond laser pulses to grating targets via SPR, resulting in the generation of bright, energetic (MeV), bi-directional electron bunches. We also compare two distinct cases satisfying resonant and non-resonant conditions using two types of gratings with different groove lines per mm as well as planar targets. In addition to, all previous results on high field surface plasmonics showing electron emission tangent to the surface^22–29^, we demonstrate electron bunch formation at angles decided by the grating parameters, suggesting tailored relativistic electron beams from periodic modulations. Incidentally, tangential emission has also been seen in plane targets with pre-plasma^37^, indicating that the enhanced emission due to surface plasmon excitation in these other studies^22–30^ may not be qualitatively different in nature. We have performed two-dimensional particle-in-cell (2D-PIC) simulations, which agrees well with experimental observation and also provide further insights into the involved physics. Apart from all previous studies^11–30^, we demonstrate emission of electron pulses at the *grating target rear,* away from other noise sources and debris at the target front. Our study also indicates efficient absorption of the laser energy by the grating structure, which should be of considerable interest for a gamut of applications, such as hard X-ray sources^10–12,38^, high harmonic generation^18–23^, fast electron and fast ion sources^37,39^, and fast electron microscopy^40^.

## Experimental setup

**Figure 1:**
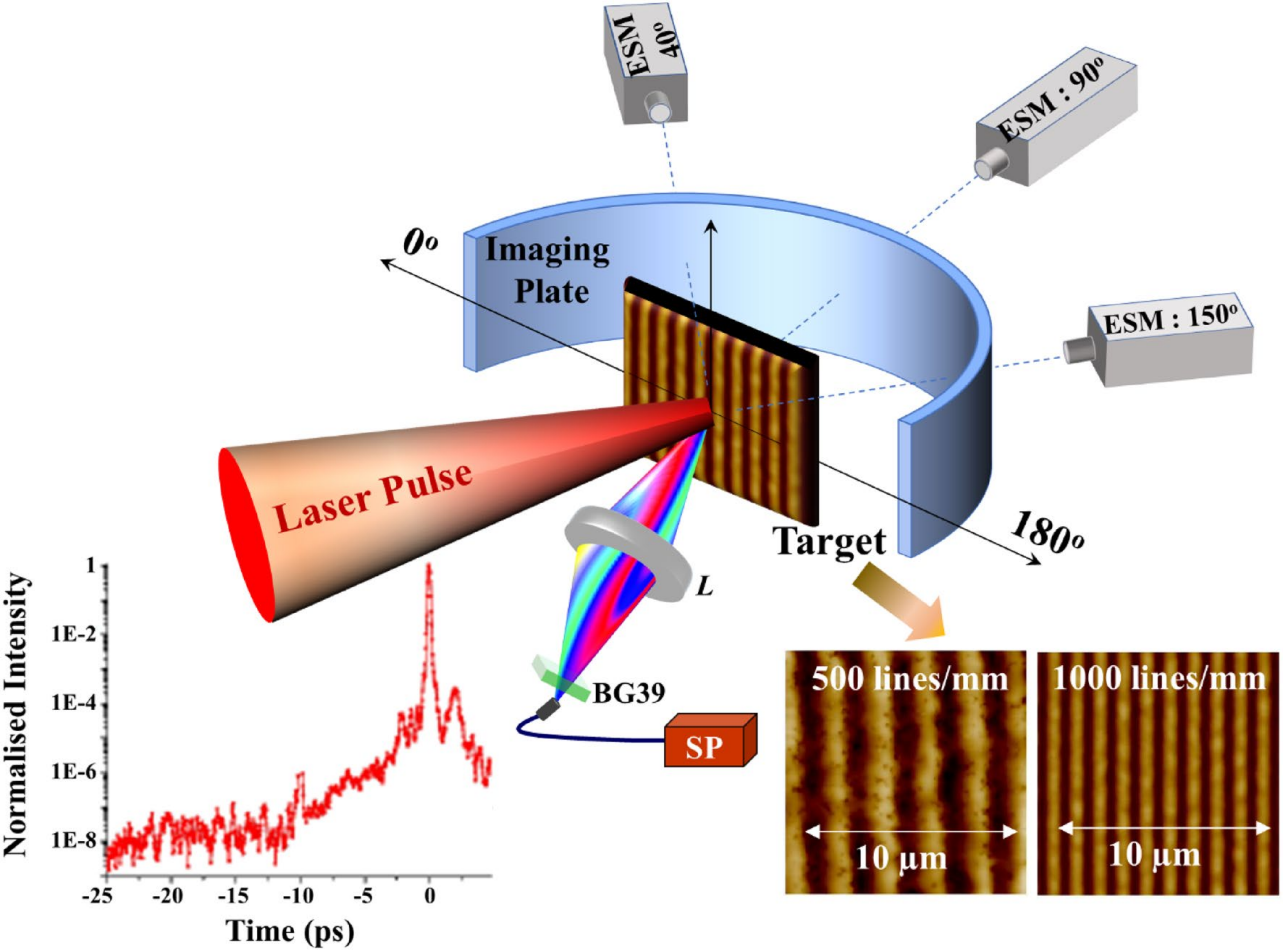
Schematic of the experimental setup. The angle of incident of interaction laser (40°) is maintained for Gr500, Gr1000, and plane Au foil. Image plate (covered with appropriate thickness of aluminium filter) is placed behind the target to measure electron angular distribution. The energies of fast electrons emerging at target rear are measured by independent electron spectrometers located along three different directions to the target (40°, 90°, and 150°). The interaction laser contrast (bottom left) and atomic field microscopic (AFM) images of the grating targets (bottom right). Plasma emission is measured at the front side of the target at specular direction. *L*: Lens, BG39: A Schott BG-39 filter, SP: spectrometer.

The experiment is performed using a 100 TW, 30 fs, 800 nm laser system at the Tata Institute of Fundamental Research (TIFR), Mumbai. These *p*-polarized laser pulses are focused on to the target using an *f*/3 off-axis parabolic mirror at 40° incidence, corresponding to a laser ellipsoidal spot size of 5 µm × 8 µm and focused peak intensity of 1 × 10^19^ W/cm^2^. A schematic of the experiment is shown in Fig. 1 (see Methods).

**Figure 2:**
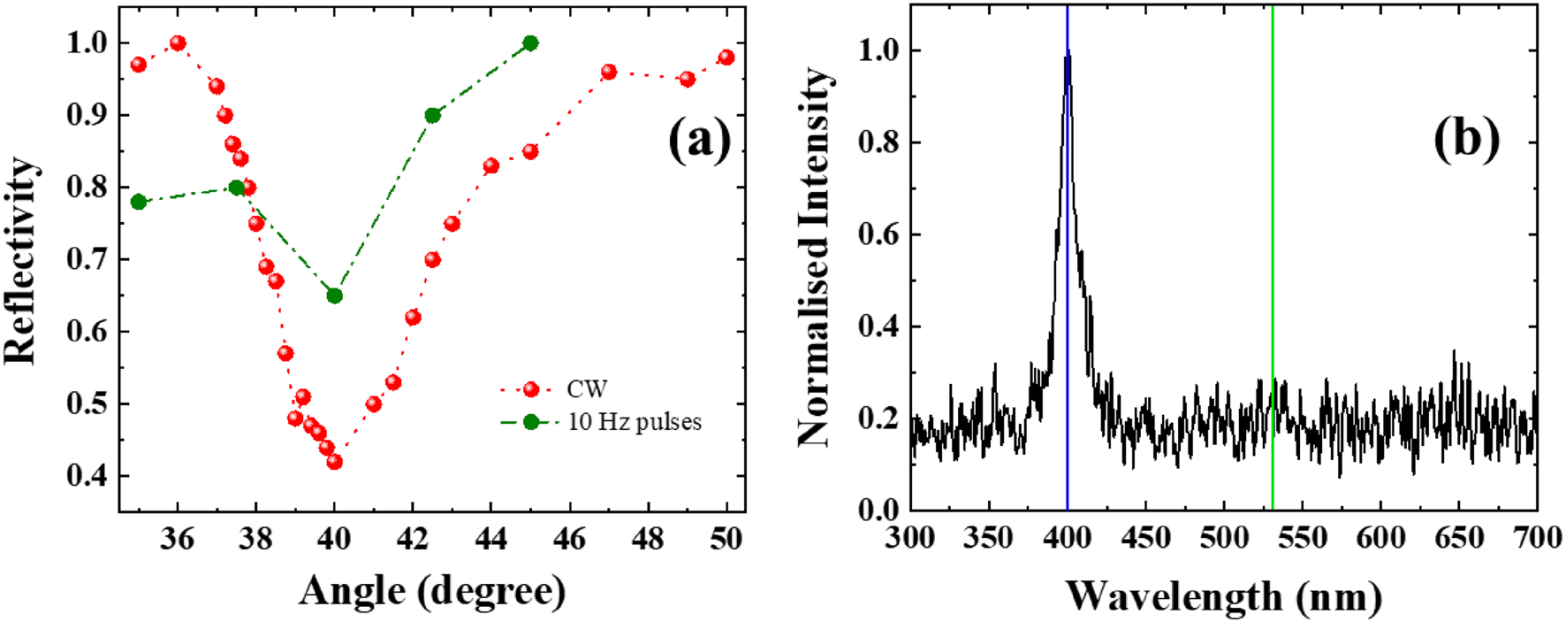
(**a**) Reflectivity of Gr500 as a function of angle of incidence. (**b**) The plasma emission for Gr500 shows generation of 2 ω (400 nm) and the absence of 3/2 ω (532 nm). The blue line indicates the position of 400 nm whereas green line indicates the position of 532 nm.

In the experiments we used two types of sinusoidal grating targets (fabricated in house) with groove density of (i) 500 lines/mm (Gr500) and (ii) 1000 lines/mm (Gr1000). Atomic field microscopic images are shown in the inset of Fig. 1. Gr500 and Gr1000 are etched to a depth of 100 nm, on 1 µm thick Au coating on 76 µm thick polyester substrate, thereby yielding total target thickness of 77 µm. We use 2.5 µm thick Au plane foil and 50 µm thick polyster substrate (mylar) as a reference target. The SPR angle of the grating is experimentally confirmed by measuring laser reflection using a Ti-sapphire oscillator at 800 nm both in the continuous-wave and mode-locked states (see Methods). This angle is further verified by 10 Hz amplified pulses at very low intensity that is less then ablation threshold. The sharp drop in reflectivity (large absorption) (SPR angle) for Gr500 (Gr1000) is observed at the angle 40° (17°) (Fig. 2a). The sharp drop in reflectivity (large absorption) (SPR angle) is observed at the angle 40° (Fig. 2a) for Gr500; and 17° for Gr1000 (Supplementary Material, Fig. S1). These results clearly show our grating target works properly in SPR angle.

The laser pulses have an intensity contrast of 4 × 10^–8^ up to 10 picosecond prior to the peak intensity, as shown in the inset of Fig. 1. The nanosecond precursor of the main laser pulse is at the level of 10^–9^. The intensity of both picosecond and nanosecond portions before the main femtosecond laser pulse is therefore below the damage threshold of the target material. An intense pre-pulse (> 10^10^ W/cm^2^) or a longer intense main interaction laser pulse (> 100 fs) can significantly deform grating target surface, and can hinder SPR condition. However, with the present generation high intensity, femtosecond lasers, SPR conditions are accessible due to typical available high contrast (> 10^–8^) and thereby lower pre-plasma levels. The energy in our pre-pulse may not be enough to produce a pre-plasma. It is well known that the three half’s harmonic (532 nm) emission caused by two plasmon decay (TPD) is a good indicator of presence of significant pre-plasma^41–44^. Our measurements (see Methods) did not detect any noticeable 532 nm emission indicating the pre-plasma is insignificant under our experimental conditions (Fig. 2b). Any residual effect of such pre-excitation is assessed from a comparison of the different grating structures (resonant and non-resonant cases). These *p*-polarized laser pulses are focused on to the grating target using an *f*/3 off-axis parabolic mirror at 40° incidence. Note that this angle satisfies SPR condition for Gr500 target only. The focal spot size is ellipsoidal shape of 5 µm × 8 µm to cover few periods of gratings for excitation of SPR.

## Results and discussion

### Plasma emission

Grating targets are irradiated using an intense laser pulse at incident angle of 40° (Fig. 1). Figure 2b shows plasma emission spectrum (in the range of 300–700 nm).

We could not detect any measurable 3/2 harmonic (532 nm) emission peak even at the highest possible intensity of 10^19^ W/cm^2^. The absence of two-plasmon decay signal (emission at 532 nm) is also clearly indicating the absence of micron-scale pre-plasma^41–44^ in the present study. This is also established in our recent measurements^45^. The presence of second harmonic (400 nm) emission, along with absence 3/2 harmonic is indicative of non-existence of significant pre-plasma of grating structure during the interaction. These facts strongly indicate the dominance of surface-plasmon resonance absorption in the experimental results described later.

### The angular distributions and energy spectra of fast electrons

**Figure 3:**
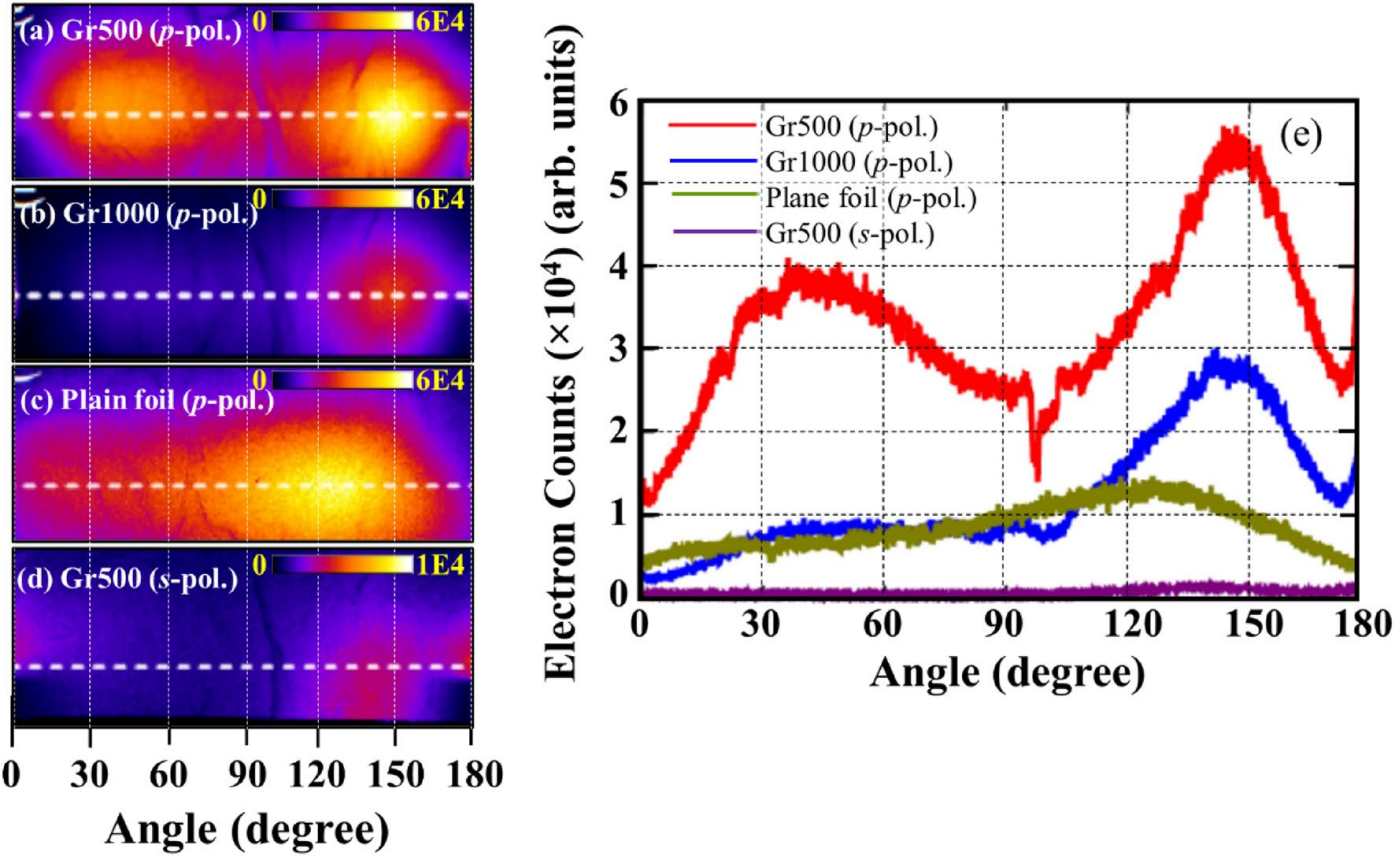
Angular distribution of electrons exiting target is measured with IP. Images of emitted electrons for grating and plane targets for (**a**) Gr500, (**b**) Gr1000, (**c**) plane Au foil, and (**d**) for Gr500 target irradiated by *s*-polarized laser. (**a**) and (**b**) show the results of single laser shot. (**c**) and (**d**) show results of 5 laser shots integration. (**e**) shows single laser shot line profiles of images for plane, Gr500, and Gr1000 targets- all under *p*-polarized irradiation, as well as that for Gr500 under *s*-polarized laser irradiation.

The angular distributions of fast electrons (see Methods) and their line profiles for Gr500, Gr1000, and 2.5 µm thick Au foil targets are shown in Fig. 3a–e. Two strong electron bunches are seen at 40° and 150° for Gr500 that satisfies the SPR condition, as shown in Fig. 3a. These two peaks are due to momentum transfer via the surface magnetic and electric fields induced at the sinusoidal grating target surface^32,46,47^. Gr1000 which does not satisfy the SPR condition, and subsequently generates only one peak around 150° (Fig. 3b), and its intensity is 2.7 times weaker than that of Gr500. In complete contrast, a plane Au foil produces electrons swaying around all directions (Fig. 3c). We also studied Gr500 with *s*-polarized intense laser pulses (Fig. 3d), which showed extremely weak electron emission. From Figs. 3c,d, one can also notice a weak electron emission component around 130°, which may have originated from *v* × *B* force along the laser axis. In terms of electron emission intensity, we see that Gr500 registers 2.7 times the electron flux of Gr1000—a non-resonant grating, and 4.3 times that from the plane Au foil (Fig. 3e).

**Figure 4:**
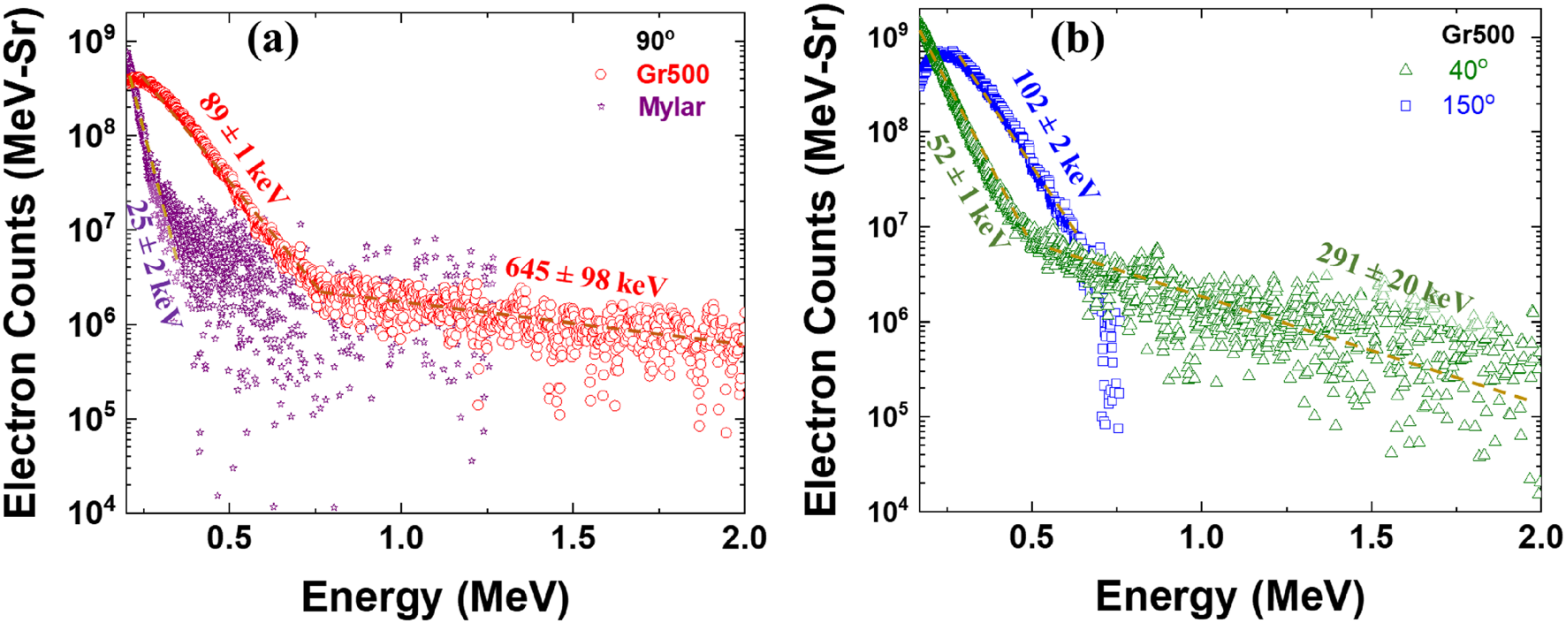
Electron spectra at (**a**) target rear normal (90°) for Gr500 and mylar substrate and (**b**) 40° and 150° at rear of Gr500.

The energy spectra of the electrons are measured with the help of electron spectrometers (ESM) (see Methods) along three different directions (Fig. 4) at the target rear. Figure 4a shows fast electron spectra measured along the target normal (90°) for Gr500 and their comparison with the data for a plane substrate (mylar). As electron spectra represent the average energy of forward going fast electrons, one can clearly notice the enhancement of the electron flux and the temperature. The electron flux for Gr500 is 6.0 times that of the plane substrate (mylar). The hot electron temperature for mylar is 25 ± 2 keV, but for Gr500 it has two components, 89 ± 1 keV and 645 ± 98 keV (Fig. 4a). Electron temperature for Gr500 enhances by factor of 3.6 compared to mylar substrate (Fig. 4a).

The fast electron temperatures are also measured for Gr500 at 40° and 150°, as fast electron bunches are observed along these directions (Fig. 3a). The angularly resolved electron spectra show (52 ± 1 and 291 ± 31) keV and 102 ± 1 keV electron temperatures at 40° and 150°, respectively (Fig. 4b). The electron flux for Gr500 increases 1.4 times for 40° and 2.4 times for 150°, compared the flux with respect to Gr500 at 90°. We also measured fast electron temperatures for Gr1000 at similar directions to that of Gr500 (40°, 90°, and 150°) (Supplementary Material, Fig. S2). The angularly resolved electron spectra show electron temperatures (75 ± 1 and 209 ± 14) keV, (63 ± 1 and 356 ± 44) keV, and 98 ± 1 keV at 40°, 90°, and 150°, respectively (Supplementary Material, Fig. S2). The electron flux for Gr1000 is same for 40° and 1.8 times for 150°, as compared the flux with respect to Gr1000 at 90°. ESM measurements affirm the SPR excitation and efficient coupling of light to fast electrons in the case of Gr500.

### The 2D3V PIC simulations

**Figure 5:**
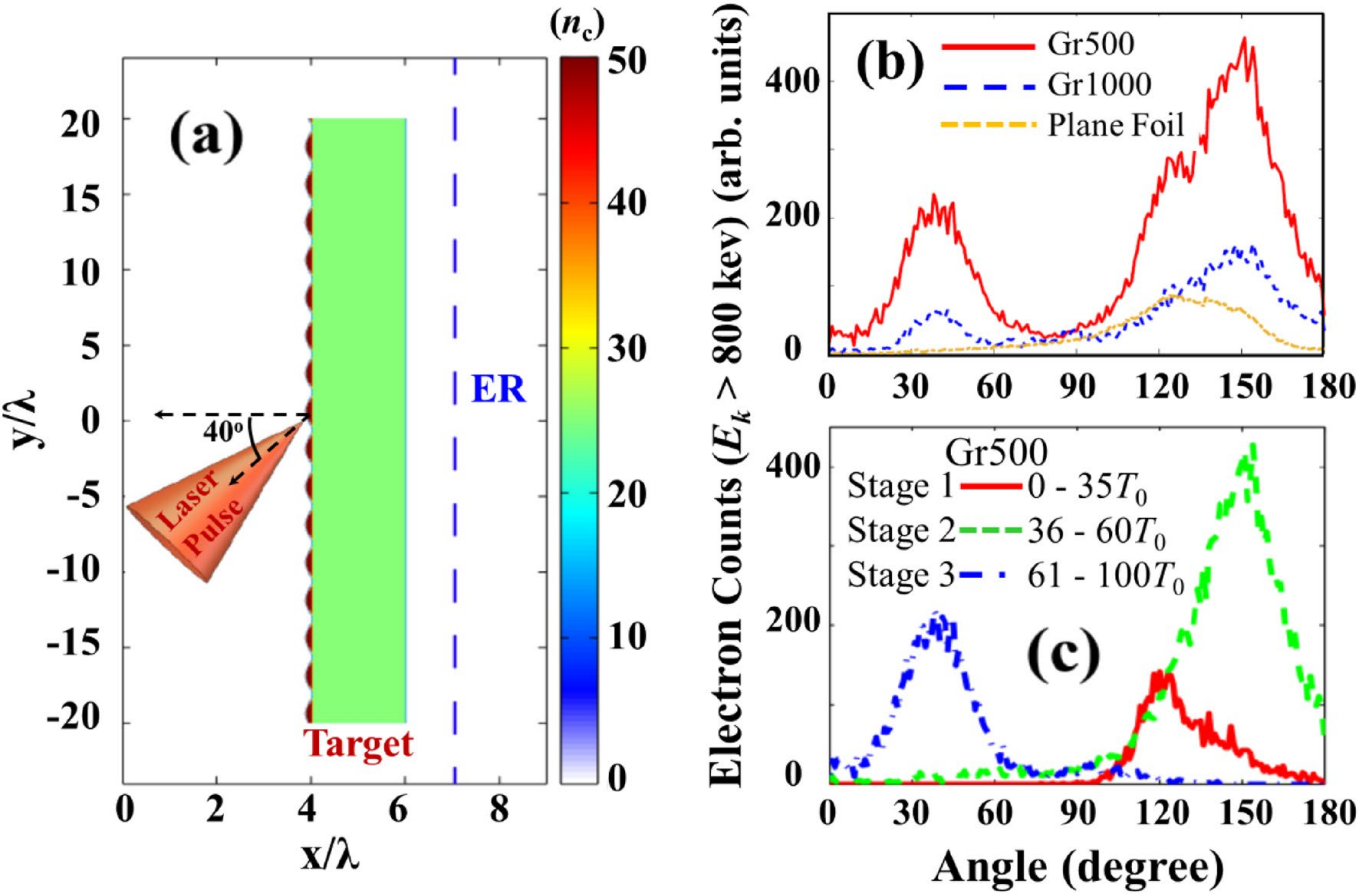
(**a**) Schematic of the simulation setup. The colorbar represents the plasma density (*n*_*c*_). ER is an electron recorder for diagnosis. The incidence angle is 40° in accordance with the experimental setup. (**b**) Angular distribution of fast electrons (E_k_ > 800 keV) recorded by ER for plane foil, Gr500, and Gr1000. (**c**) Electron emission detected during all three stages of laser interaction time profile for Gr500. Stage 1: 0–35 *T*_0_ (red curve—representing laser rising edge); Stage 2: 36–60 *T*_0_ (green curve—after the laser peak until the upper-forward electrons fade away), Stage 3: 61–100 *T*_0_ (blue curve—after the Stage 2 until the all the detectable electrons vanish). The moment in each stage here is several *T*_0_ later than the laser loading time due to the propagation delay (i. e., *t* = 30 *T*_0_ is the moment at which the laser peak impinges on the target. However, about additional 5 *T*_0_ is required for electrons propagating from the target surface to ER, hence the corresponding time for electrons being detected is *t* = 35 *T*_0_).

After establishing the role of the surface plasmons in the experiment, we verify the fast electron dynamics using PIC simulations. A schematic of the simulation is shown in Fig. 5a. The 2D3V PIC simulations are carried out using the code OSIRIS^48^ (see Methods). We compare the two types of grating targets used in experiment, Gr500 and Gr1000, to ascertain the resonant acceleration process of fast electrons. As shown in Fig. 5a, the grating layer is 100 nm thick sinusoidal Au^10+^ with 50*n*_*c*_ plasma density. The laser front reaches the focal point at *t* ~ 16*T*_*0*_, where *T*_*0*_ is the laser period. An electron recorder (labelled by ER in Fig. 5a is set at one- λ behind the target to diagnose fast electrons and tag them; only forward-going electrons with *E*_*k*_ > 200 keV are recorded, and counted once. The backward moving electron counts, if any, are eliminated from the recorder. The emission angle is calculated by *θ* = arctan (*p*_*y*_/*p*_*x*_). The simulated angular distributions of fast-electron emission from plane foil, Gr500, and Gr1000 targets are shown in Fig. 5b, which agree well with the experimental observation. Fast electrons are mainly emitted in bunches along two directions, 40° and 150° (Fig. 3e). The number of energetic electrons for Gr500 is about 2.7 times higher than the number for Gr1000. These results clearly confirm the enhancement of electron beam under SPR condition. Also, note that the second peak around 40° is observed in Gr1000, whereas experiment shows broad peak around 40° mainly due to the low electron flux on to the IP.

To understand the effects of grating structure on electron acceleration and emission, we analyse the electron dynamics systematically. Three distinct stages of electron motion and thereby electron angular distribution is found. Figure 5c shows the electron emission detected at the Stage 1 at the rising edge of the laser pulse, before the laser peak arriving at the target surface (0–35 *T*_0_); the Stage 2 after the laser peak until the upper-forward electrons fade away (36–60 *T*_0_); and the Stage 3—after Stage 2 until all the detectable electrons vanish (61–100 *T*_0_). During these three stages fast electrons are emitted at 120°, 150°, and 40° directions, consequently. As the number density of electrons is relatively weak during the Stage 1 (red curve in Fig. 5c), the dominant electron emission peaks are seen at 150° (green curve in Fig. 5c) and 40° (blue curve in Fig. 5c). The angular distribution of electron emission (Fig. 5b) thus matches very well with that from the experiments (Fig. 3e). Our simulation shows that electron emission along the laser propagation direction is suppressed, which is attributed to the increased surface fields and the modulation of the *p*-polarized laser pulse by the grating surface. The *v* × *B* motion of electrons is heavily affected and suppressed. This is consistent with experimental observations. This also establish the relevance of grating geometric effects along with SPR.

**Figure 6:**
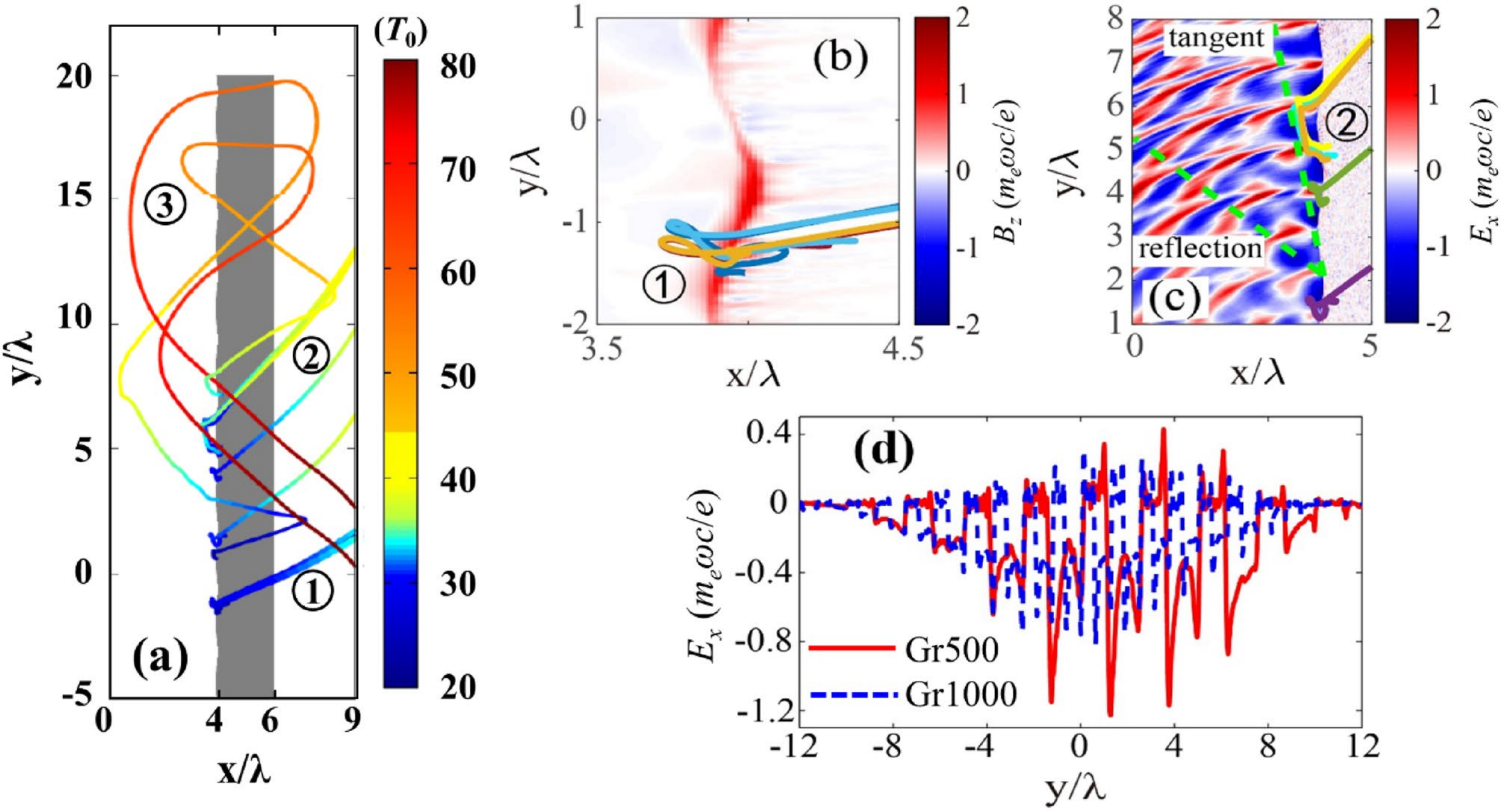
(**a**) Trajectories of fast electrons in the three distinguished stages of laser pulse irradiation. The colorbar represents the simulation time (*T*_0_). Stage 1: 0—35*T*_0_, Stage 2: 36—60*T*_0_, Stage 3: 61—100*T*_0_. (**b**) Quasi-static magnetic field *B*_*z*_ at *t* = 25 *T*_0_ and two selected electron trajectories around this time. (**c**) The transient field *E*_*x*_ at *t* = 35 *T*_0_ and four selected electron trajectories around this time, where an electromagnetic field component is shown to propagate along the tangent direction in addition to the reflected laser (green dashed line). (**d**) Surface electric field *E*_*x*_ for Gr500 and Gr1000 at *t* = 30*T*_*0*_, the moment at which the laser peak impinges on the target. *B*_*z*_ and *E*_*x*_ are normalized by *m*_*e*_*ωc*/*e*.

Next, we retrospectively track some trajectories of electrons, as shown in Fig. 6a. Electrons are initially pulled out of the target and then gets injected back. Electron dynamics in Stage 1 is very similar to the Brunel heating mechanism^35^. They are first pulled out by the electric field of laser and pushed back in the next half period, as shown in Fig. 6b. In the meanwhile, there is quasi-static magnetic field generated on the surface due to the grating structure. Through the surface magnetic field^49^, fast electrons are deflected at an angle of *θ* ~ *eBL*/*γm*_*e*_*c* ~ 30° (120° to the tangent), where *B* ~ *a*_*0*_*m*_*e*_*ω*/2*e* is the estimated magnetic field and *L* = *c*/*ω* is the thickness of surface magnetic field in Fig. 6b.

**Figure 7:**
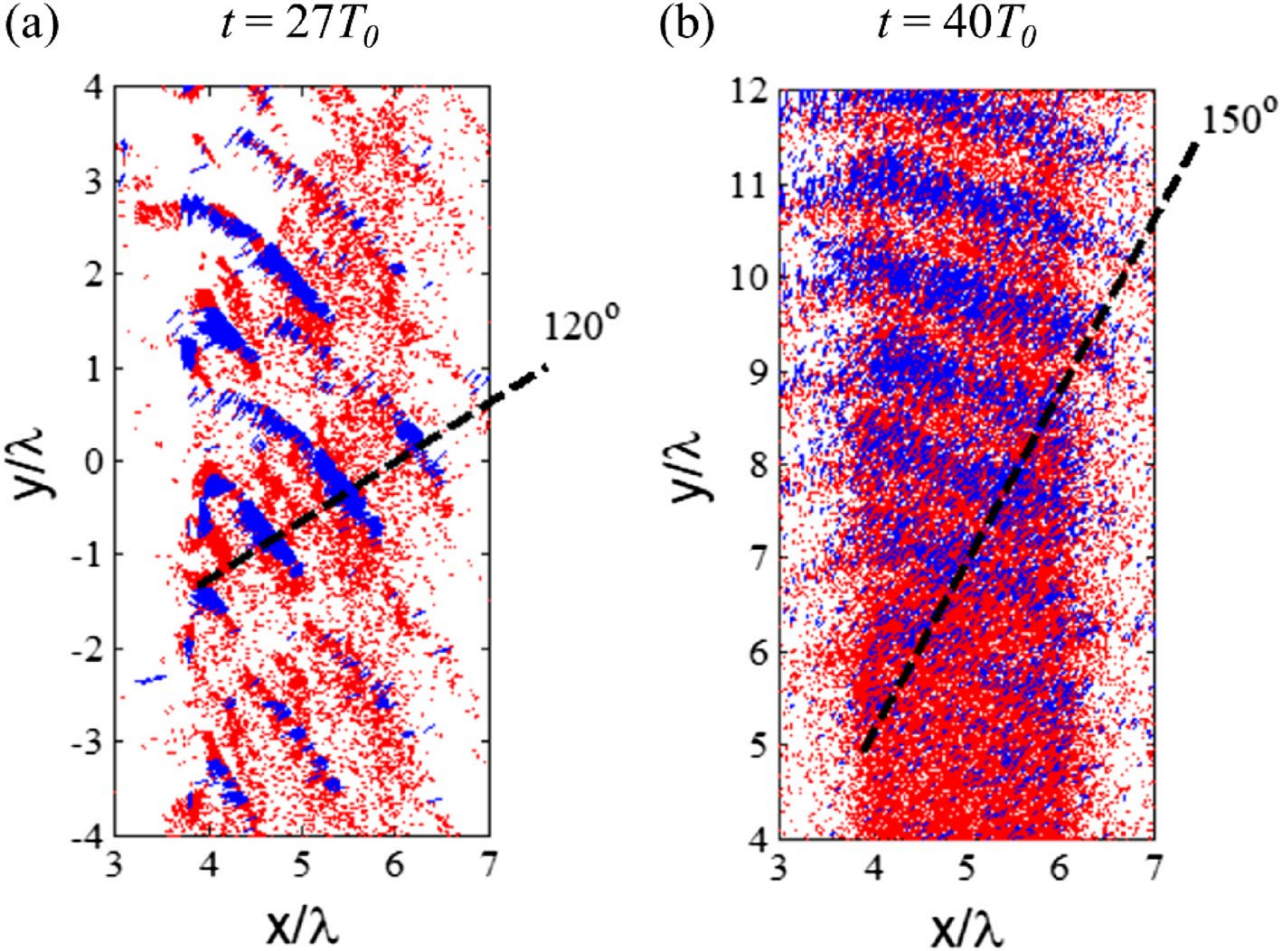
The transient electron emission patterns with the Gr500 target at (**a**) *t* = 27 *T*_0_; and (**b**) *t* = 40 *T*_0_.

In Stage 2, the surface plasma waves begin to play a major role in fast electron generation. Figure 6c shows the instantaneous electric field *E*_*x*_ at *t* = 35 *T*_0_. One can see an electromagnetic field component propagating along the tangent direction in addition to the reflected laser (green dashed line Fig. 6c). The electrons which are initially accelerated by the laser field through the Brunel mechanism gets further accelerated by the surface electromagnetic waves; however, the acceleration is soon de-phased as the phase velocity of surface fields is larger than the electron speed. In the present case, the final electron energy is around 200 to 800 keV with the velocity about 0.9 to 0.96 c. After separating from the surface electromagnetic fields, the electrons are pushed forward by the electrostatic field *E*_*x*_ (~ 2 × 10^12^ V/m) and meanwhile deflected by the magnetic field *B*_*z*_ (~ 54 MG) built around the target. The motion equation of these electrons is *dθ*/*dx* = *eB/p*, where *p* = *γβm*_*e*_*c* is the electron momentum and *γ* = *eE*_*x*_*x*/*m*_*e*_*c*^2^ + 1 is the Lorentz factor. It is found that the acceleration length is approximately *x* = *H* + *L*, where *H* = 100 nm is the groove depth of grating. Figure 7 shows the electron density flux for Gr500 at *t* = 27 *T*_0_ (Fig. 7a) and 40 *T*_0_ (Fig. 7b). Low energy electrons are represented by the red points (200 to 800 keV), whereas energetic electrons are represented by blue points (> 800 keV). The energetic electrons move 120*◦* at the early stage, and then changes their direction towards 150*◦* at the later time. Hence, one can estimate the deflection angle in Stage 2 to be θ ~ 60° (150° to the tangent). Although the energetic electron emission angle changes with time, the emission pattern still shows emission frequency close to incident laser frequency. This corresponds to the surface plasmon wave or vacuum heating mechanism.

Electron tracks in Stage 3 appear complicated but also unambiguous and explainable. The origin is no longer the Brunel electrons. In the laser falling edge, the reflected laser is stronger than the incident one. Some electrons are directly accelerated by the reflected laser. However, a part of these electrons will be pulled back by the sheath electric field and undergo approximately specular reflections at each side. Since the target is of finite dimension, these fast electrons will be bounced back by the sheath electric field and finally emit from the rear of the target where the sheath electric field is very weak after the laser impinging. As discussed above, the electron acceleration using grating targets is finally determined by the surface electrostatic field *E*_*x*_ and magnetic field *B*_*z*_. Such fields at the front surface will be highly enhanced under the SPR condition. The fields at the rear surface will be indirectly enhanced due to the larger electron flux and current.

**Figure 8:**
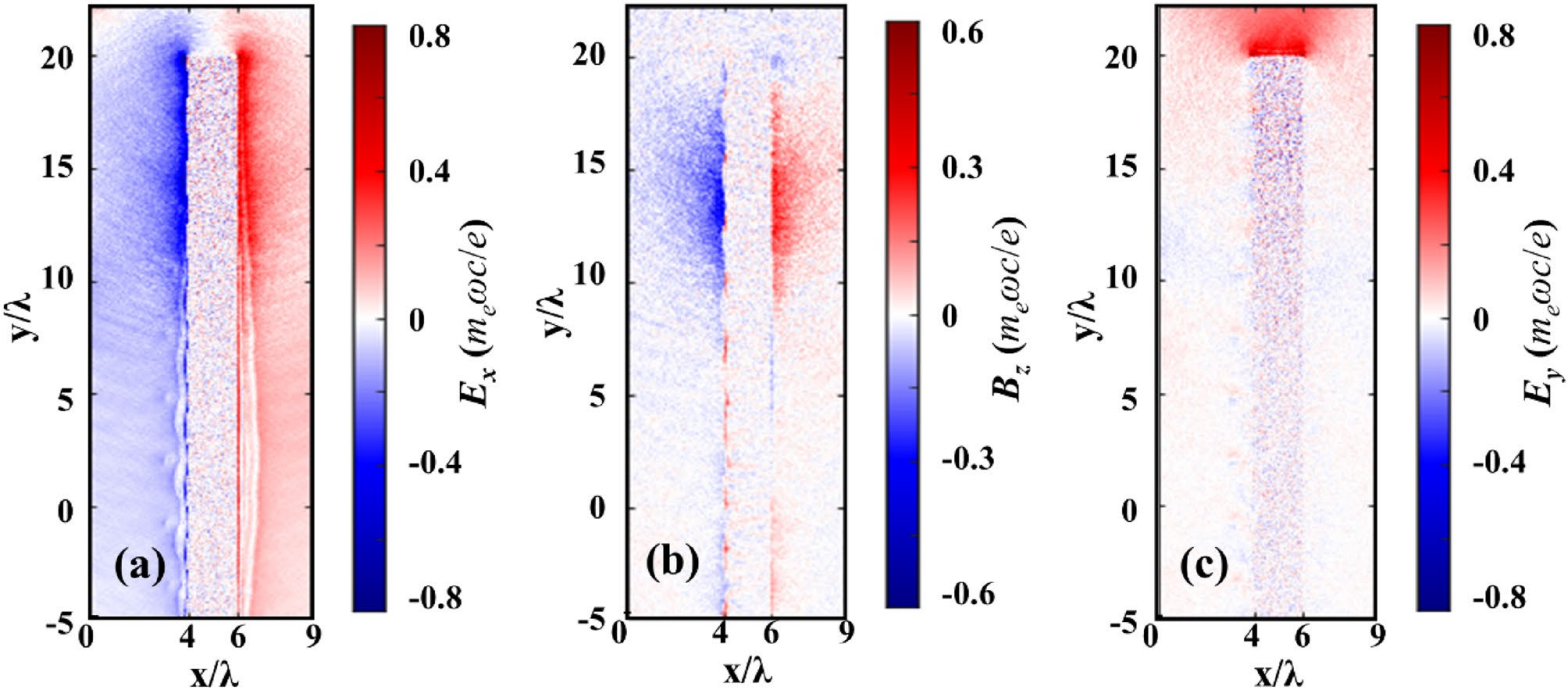
Quasi-static electromagnetic fields (**a**) *E*_*x*_, (**b**) *B*_*z*_, and (**c**) *E*_*y*_ at *t* = 60 *T*_0_ for Gr500. The electric and magnetic fields are normalized by *m*_*e*_*ωc*/*e*.

Two typical trajectories for such electrons’ emission at this stage are shown in Fig. 6a. The electrons firstly experience transverse deflection at the rear side of the target and then transmit through the target due to the sheath field. In the front side, they experience similar process and finally go through the target with an emission angle of 40°. Such transverse deflection results from the combined effects of the quasi-static electromagnetic fields *E*_*x*_ and *B*_*z*_ around both the front and rear sides of the target and is related to the *E* × *B* drift, which can be clearly seen from the typical trajectory (Fig. 6a) and the fields at rear side (Fig. 8a–c). The fields are at a fixed time (*t* = 60 *T*_0_), so the integration effects cannot be clearly seen. However, they are relatively slowly varying fields. The combined effects of the quasi-static electric and magnetic fields lead to the electron reflection. These results are consistent with earlier simulations by Bigongiari et al*.*^26^ who showed that (a) the surface plasmon wave can significantly enhance the quasi-static magnetic fields at the surface; and (b) these intense localized magnetic fields can determine the divergence and flux of the energetic particles in the beam. Figure 6d compares the *E*_*x*_ fields at the laser peak moment for grating targets with different grove spacing. The *E*_*x*_ for Gr500 is 1.5 times larger than *E*_*x*_ for Gr1000. This leads to the increase of electron energy and number simultaneously, and thus 2.3 times enhancement of flux for Gr500, which are similar as those observed in the experiments shown in Fig. 3e.

## Summary and conclusions

We demonstrate enhanced generation of two distinct fast electron bunches exiting from grating targets, due to efficient energy transfer by femtosecond, high contrast, relativistic intense laser pulses to grating structure. We demonstrate the role of surface plasmon excitation and show the emergence of a fast electron beam at the angle determined by both grating and laser incidence parameters. Our results are fully explained by 2D-PIC simulations, where three stages of electron emission are found.

Significant increase of fast electron generation would be of critical importance to applications in a variety of areas of science and technology. There are many approaches to generate the electron beams from the solids during intense laser-plasma interactions^50–60^. From the point of view of the applications, it is educative to compare electron beams from solids at relativistic laser intensity excitation levels with those from laser wakefield acceleration (LWFA) in plasmas^50^. The energy of electron beams from solids is typically in the few to tens of MeV, while those from LWFA cover the range keV to GeV energy per particle^51–53^. The directionality of the electron beams from a solid surface depends on the mechanism of absorption and can be along the laser, in the specular direction or at an angle determined by the surface modulation, as we have illustrated in the present study. The LWFA electrons are highly directional along the laser and the divergence is as low as sub-mrad^50^. It can be controlled by adjusting the densities of the plasma, guiding schemes and counterpropagating pulses. The duration of electron pulses in both schemes can be in the femtosecond regime, though the dispersion on propagation may be larger in the beams from solids, due to the higher charge repulsion^50^.

The above comparison is useful while choosing one of the schemes for a practical application. For high flux applications, solid plasmas are an obvious choice, while for highly relativistic beams, LWFA is preferable. Another interesting point is the further use of either for the generation of electromagnetic radiation (both hard and soft). The beams from solids are known to generate high energy (µJ to mJ) terahertz radiation^61–63^, the LWFA beams can generate radiation efficiently in the X-ray region via the betatron oscillation mechanism^64^.

Our study will provide a benchmark for further exploration of directional, relativistic, ultrashort electron bunch formation from surface structured targets.

## Methods

### Measurement of surface plasmon resonance angle for grating targets

Surface plasmon is an electron oscillation at the dielectric-metal interface and light energy is absorbed efficiently when the wavenumber of input light matches that of the surface plasmon at a certain incidence angle (the resonance angle). This angle is calculated from $\mathrm{sin}{\theta }_{sp}+\frac{\lambda }{d}= \sqrt{\frac{\varepsilon }{\left(\varepsilon +1\right)}}$ where, *θ*_*sp*_, λ, *d*, and ɛ indicate the resonance angle, the wavelength of the laser, the grating spacing, and the permittivity of target material, respectively^65^. For plasma on the grating surface, we can rewrite this as,

$$\sin \theta_{sp} + \frac{\lambda }{d} = \sqrt {{{\left( {1 - \frac{{n_{e} }}{{n_{c} }}} \right)} \mathord{\left/ {\vphantom {{\left( {1 - \frac{{n_{e} }}{{n_{c} }}} \right)} {\left( {2 - \frac{{n_{e} }}{{n_{c} }}} \right)}}} \right. \kern-\nulldelimiterspace} {\left( {2 - \frac{{n_{e} }}{{n_{c} }}} \right)}}},$$

where *n*_*e*_ and *n*_*c*_ indicate the electron density and the critical density (*n*_*c*_ ~ 1.73 × 10^21^ cm^-3^ at λ = 800 nm)^66^. This SPR angle of each grating is ascertained by measuring laser reflection using a Ti–sapphire oscillator at 800 nm both in the continuous-wave and mode-locked states. This angle is further verified by 10 Hz amplified pulses at very low intensity (Fig. 2a).

### Measurement of plasma emission

Grating targets are irradiated using the TIFR 100 TW, 30 fs, 800 nm laser system. These *p*-polarized laser pulses are focused on to the target using an *f*/3 off-axis parabolic mirror at 40° incidence (Fig. 1), corresponding to a laser ellipsoidal spot size of 5 µm × 8 µm and focused peak intensity of 1 × 10^19^ W/cm^2^. The plasma emission is captured with an optical-fibre coupled to a UV–VIS-NIR spectrometer (Avantes-2048, spectral range 200–1100 nm) on the front side of the Gr500 target (Fig. 2b). A Schott BG-39 filter (transmission window: 300–700 nm) is used in front of the fibre to avoid spectrometer saturation and damage from the fundamental 800 nm laser (ω).

### Measurement of angular distributions and energy spectra of fast electrons

Angular distributions of electrons propagating forward and exiting target are measured with imaging plates (IPs)^67^ (FUJI Film, BAS-SR 2025) of a size [20 mm (width) × 87 mm (height)], placed 60 mm behind the target and covering the angular range from 0 to 180° (Fig. 1). The IPs are covered with aluminium filters of different thickness to prevent exposure to X-rays, direct laser, plasma emissions, and ambient light in the range from 0 to 180°. An Al filter of 11 µm thickness is used for Au foil target, while another Al one of 165 µm thickness is used for Gr500 and Gr1000 targets. The corresponding electron energy threshold for detection of electrons on IP is 10 keV for 11 µm Al filter, and 175 keV for 165 µm Al filter, respectively. The energies of fast electrons emerging at target rear are measured by independent electron spectrometers located along three different directions to the target, at focused laser peak intensity of 1 × 10^19^ W/cm^2^. Each spectrometer has a 0.1 Tesla magnetic field with an IP as the detector. The measurable range of energies in these spectrometers is 0.1–7.0 MeV.

### 2D3V PIC simulations using the OSIRIS^48^ code

To execute the simulations the polyester substrate is geometrically scaled down with 2λ thickness and 40λ width. The polyester consists of C^4+^ and H^1+^ with 25*n*_*c*_ plasma density. The grid size is set to be *d*_*x*_ = *d*_*y*_ = λ/64, which are precise enough to resolve the plasma skin depth. 64 particles are included in each grid cell and all of the particles are mobile. The *p*-polarized laser pulse with electric field envelope *E* = *E*_*0*_ exp(− *r*^2^/*w*^2^) sin^2^(π*t*/2τ) [λ = 800 nm, *E*_*0*_ = 8.7 × 10^12^ V/m, τ ~ 30 fs, w ~ 5λ), where τ is the laser duration and w is the laser waist, irradiates the grating surface with 40° incident angle. A reduced target thickness has been used in the simulation compared to the experiment, since it is extremely difficult to perform simulations as per the experimental target thickness as they are presently beyond our computational capabilities and are highly expensive; and hopefully will overcome those in our future studies.

## Supplementary Information

Supplementary Information.

## Data Availability

The data used to support the findings of this study are available from the corresponding author upon request.
