# Supplementary material for: Luminous, relativistic, directional electron bunches from an intense laser driven grating plasma
Source: Sci Rep. 2022 Oct 7;12:16818. doi: 10.1038/s41598-022-21210-7 (PMC9546899; doi:10.1038/s41598-022-21210-7)
Supplement: Supplementary file 1 — Supplementary Information. [file 41598_2022_21210_MOESM1_ESM.docx]

**Supplementary Material for**

**Luminous, Relativistic, Directional Electron Bunches from**

**an Intense Laser Driven Grating Plasma**

Amit D. Lad^1^, Y. Mishima^2^, Prashant Kumar Singh^1^, Boyuan Li^3^, Amitava Adak^1^, Gourab Chatterjee^1^, P. Brijesh^1^, Malay Dalui^1^, M. Inoue^4^, J. Jha^1^, Sheroy Tata^1^, M. Trivikram^1^, M. Krishnamurthy^1^, Min Chen^3,5^, Z. M. Sheng^3,5,6^, K. A. Tanaka^2,7^, G. Ravindra Kumar^1^, & H. Habara^2,*^

^1^Tata Institute of Fundamental Research, 1 Homi Bhabha Road, Colaba, Mumbai 400005, India.

^2^Graduate School of Engineering, Osaka University, Suita, Osaka 5650871, Japan.

^3^Key Laboratory for Laser Plasmas (Ministry of Education), School of Physics and Astronomy, Shanghai Jiao Tong University, Shanghai 200240, China.

^4^Faculty of Science and Engineering, Setsunan University, Neyagawa, Osaka 5728508, Japan.

^5^Collaborative Innovation Center of IFSA (CICIFSA), Shanghai Jiao Tong University, Shanghai 200240, China.

^6^SUPA, Department of Physics, University of Strathclyde, Glasgow G4 0NG, United Kingdom.

^7^Extreme Light Infrastructure: Nuclear Physics, 30 Reatorului, Magurele – Bucharest 77125, Romania.

^*^Correspondence and requests for materials should be addressed to H.H. (email: [habara@eei.eng.osaka-u.ac.jp](mailto:habara@eei.eng.osaka-u.ac.jp))

**Reflectivity measurement for Gr1000:**

| 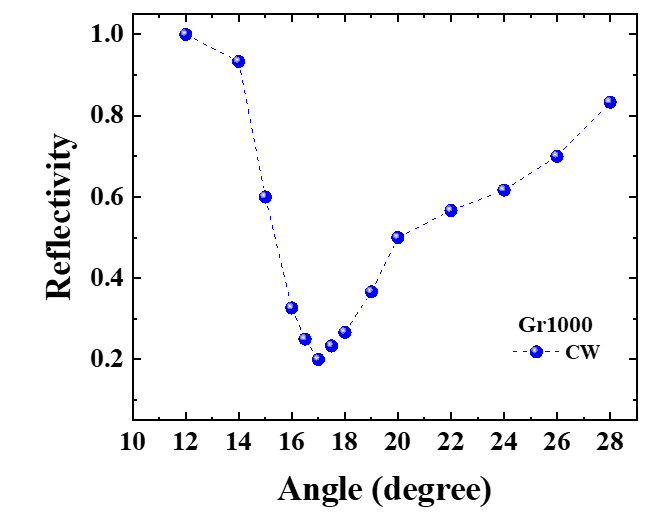 |
| --- |
| Fig. S1. Reflectivity of Gr1000 measured with CW laser as a function of angle of incidence. |

**Energy spectra of fast electrons for Gr1000:**

| 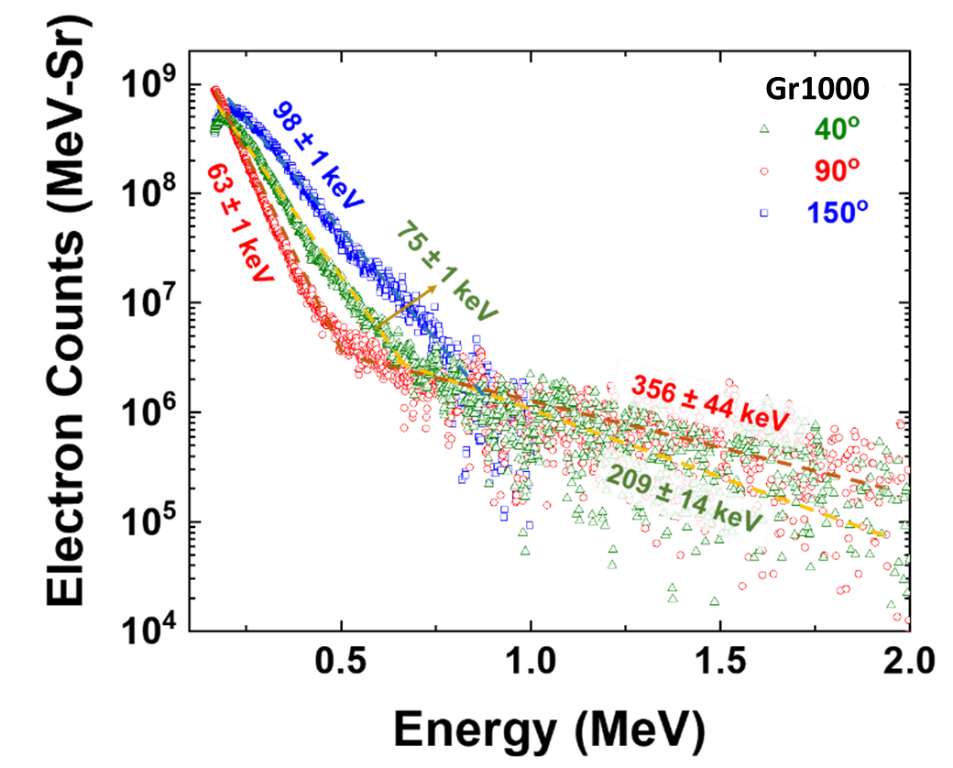 |
| --- |
| Fig. S2. Electron spectra at 40^o^, 90^o^, and 150^o^ at rear of Gr1000. |
